# Latent profiles of innovative behavior among hemodialysis nurses and their association with artificial intelligence literacy: a cross-sectional study

**DOI:** 10.3389/fmed.2026.1822694

**Published:** 2026-06-30

**Authors:** Tong Li, Li He, Hao-Tian Zheng, Hui Chen, Li Liu, Ying-Jun Zhang, Lin Chen

**Affiliations:** Department of Nephrology, Hemodialysis Center, West China Hospital, Sichuan University/West China School of Nursing, Sichuan University, Chengdu, Sichuan, China

**Keywords:** artificial intelligence literacy, cross-sectional study, innovative behavior, latent profile analysis, nurse

## Abstract

**Background:**

The growing hemodialysis patient population has substantially increased nurses’ workload in this specialty. While innovative behaviors are known to enhance both hospital operational efficiency and nursing competencies, research focusing on hemodialysis nurses’ innovation remains limited. Notably, no studies have yet examined the potential relationship between this workforce’s innovative behaviors and their artificial intelligence (AI) literacy.

**Objective:**

This study aimed to identify latent profiles of innovative behavior among hemodialysis nurses, examine their associations with sociodemographic factors and artificial intelligence literacy, and explore predictors of profile membership.

**Methods:**

A cross-sectional survey was conducted among 493 hemodialysis nurses in Sichuan Province, China. LPA was used to determine the potential categories of innovative behavior of hemodialysis nurses, and Pearson correlation was used to analyze the relationship between innovative behavior and AI literacy. Binary Logistic regression was used to analyze the influencing factors of different categories of innovative behaviors.

**Results:**

The latent profile analysis of innovative behaviors of hemodialysis nurses revealed that the second potential model was the best model (AIC = 10902.747, BIC = 11032.963, entropy = 0.940). The two potential categories were as follows: “high innovative behavior-transcendence” (56%) and “low innovative behavior-conservative” (44%). Correlation analysis showed that there was a positive correlation between innovative behavior and AI literacy of hemodialysis nurses (*r* = 0.373, *P* < 0.01). Multivariate analysis showed that educational background (OR = 0.500, 95%CI: 0.264∼0.949, *P* = 0.034), position of work (OR = 2.074, 95%CI: 1.115∼3.859, *P* = 0.021) and AI literacy level (OR = 1.075, 95%CI: 1.053∼1.096, *P* < 0.001) were the influencing factors of innovative behavior.

**Discussion:**

The identification of two distinct innovative behavior profiles reveals substantial heterogeneity among hemodialysis nurses. The significant positive correlation between AI literacy and innovative behavior highlights the potential of AI competency development as a novel pathway to stimulate nursing innovation. Moreover, educational background and job position as key influencing factors imply that systematic continuing education and expanded leadership roles may effectively elevate innovation, especially for nurses in the “low innovative behavior-conservative” group. These findings provide actionable guidance for nursing managers to incorporate AI literacy training into professional development programs and to design targeted support strategies aimed at cultivating a more proactive, innovative nursing workforce.

## Introduction

At present, nurses account for up to 50% of the global medical and health workers (1). With the rapid development of technology and medical field, the importance of nursing in the health field has become more and more significant. In order to keep up with the pace of the medical industry, nurses need to constantly innovate and learn. Nursing innovation refers to the ability to actively seek and develop new methods, technologies, and tools to promote health, prevent disease, improve the quality of patient care, and facilitate teamwork through reasonable channels of support (2, 3). Studies have shown that nursing innovation has an important impact on organizational performance and the prognosis of patients. Therefore, the influencing factors of inspiring innovative ideas and promoting innovative behaviors of nurses are worth exploring. At present, society has entered the digital era. While gradually covering the medical field, artificial intelligence (AI) may also provide new ways and opportunities for the innovative behavior of nurses. Artificial intelligence is broadly defined as the ability of computer systems to perform tasks that normally require human intelligence, such as reasoning, communication, and decision-making (4). As early as 1985, there were data on artificial intelligence in nursing (5). At present, the application of artificial intelligence in the field of nursing is mainly in mobilization support, drug dosage adjustment, and administration (6). Moreover, Artificial Intelligence-driven tools are developing rapidly in learning pathways such as virtual simulation and big data analysis (7), and nurses are likely to amplify their creativity through personalized learning, promoting interactive problem solving and optimizing decision making.

In recent years, with the aging of the population and the increasing prevalence of diabetes and hypertension, the number of hemodialysis patients is also increasing year by year (8). By the end of 2024, the number of hemodialysis patients in China reached 1 million. This means that there will be an increasing number of people working in hemodialysis care. Due to the particularity of nursing and treatment, hemodialysis nurses not only need to operate dialysis machines independently, deal with various emergencies and dialysis-related complications of patients, but also assume a variety of roles such as technicians and educators (9). Given the particularities of hemodialysis nurses in the context of dialysis practice, promoting innovative behaviors of this group is beneficial to promote the development of the dialysis field. However, innovative behavior is a dynamic, complex and multi-stage process (10), and the innovative behavior of Chinese hemodialysis nurses is still at a moderate level under high-intensity and difficult work environments (11). At present, there is no survey on the artificial intelligence literacy of hemodialysis nurses, but the survey of operating room nurses shows that the level of artificial intelligence literacy of nurses is also at a medium level (12). Wu et al. (13) mentioned “scientific research and innovation ability” as one of the core competence indexes of blood purification nurses in the evaluation index system of core competence of blood purification nurses formulated according to China’s national conditions. Therefore, the innovative behavior and artificial intelligence literacy level of hemodialysis nurses deserve attention. This study aims to determine the potential characteristics of innovative behavior of hemodialysis nurses using latent profile analysis through the survey of hemodialysis nurses, and to explore the influence of socio-demographic factors and artificial intelligence literacy on innovative behavior. Specifically, the following research questions were addressed: (a) What distinct profiles of innovative behavior can be identified among hemodialysis nurses through latent profile analysis? (b) Do sociodemographic and work-related characteristics differ across the identified profiles? (c) Is there a significant relationship between innovative behavior and AI literacy among hemodialysis nurses? (d) Which factors (sociodemographic variables and AI literacy level) independently predict membership in higher versus lower innovative behavior profiles?

## Materials and method

### Participants

We conducted a convenience sampling survey of nurses engaged in hemodialysis work at hospitals across all levels in various provinces and cities of Sichuan, China from April to May 2025. The inclusion criteria for the participants are as follows: (a) nurse practice certificate of the People’s Republic of China; (b) at least 1 year of hemodialysis-specific nursing experience and (c) Voluntary participation in this survey. Exclusion criteria: (a) Never engaged in hemodialysis nursing work.

**FIGURE 1:**
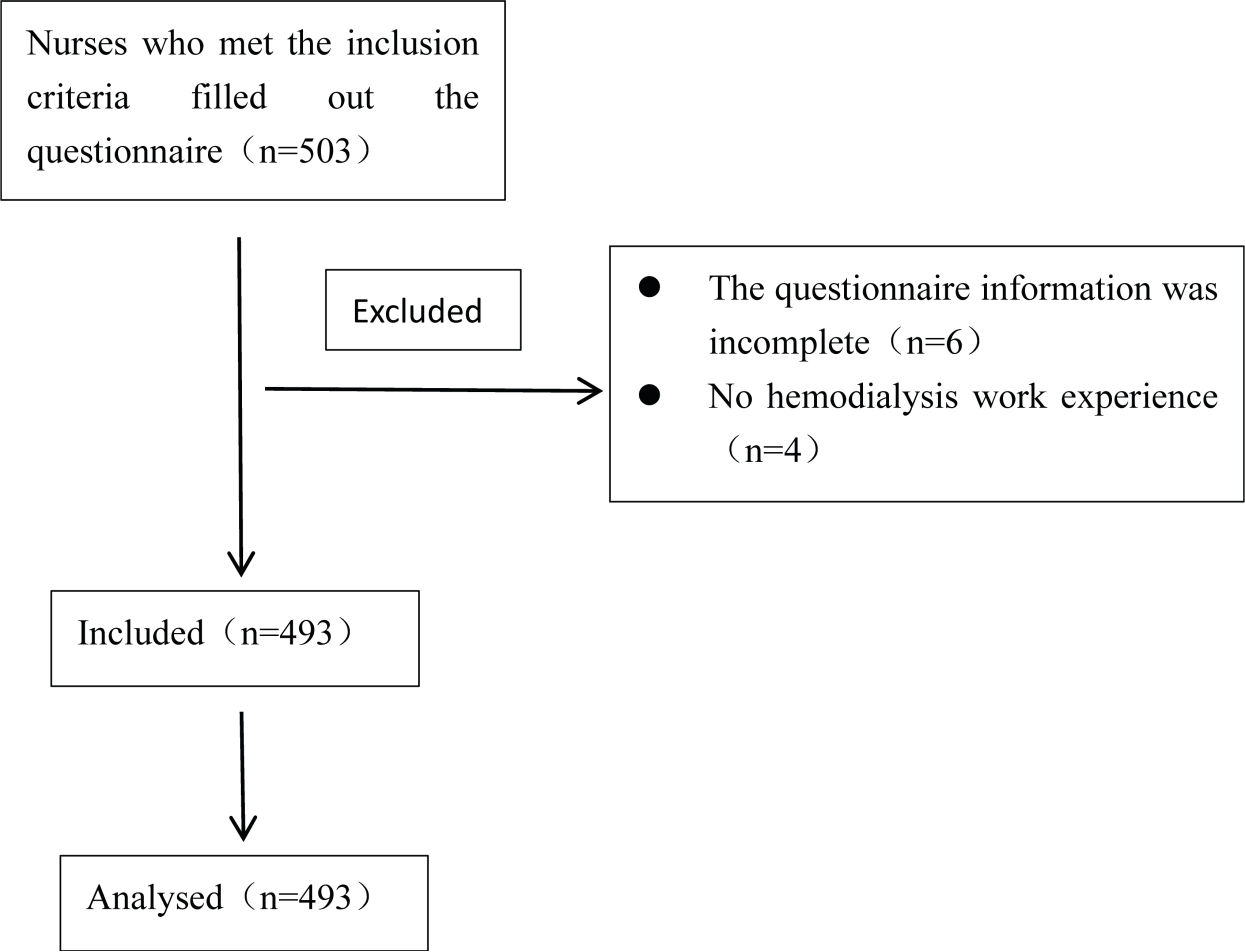
Flowchart.

The sample size was calculated according to Kendall’s guidelines (14). Sample size is recommended to be at least 10-20 times the number of independent variables. For the primary multivariate logistic regression, the number of independent variables to be examined was approximately 22. Considering a 20% attrition rate, the sample size was suggested to be between 264 and 528. In this study, a total of 503 questionnaires were distributed, 10 unqualified questionnaires were excluded, and a total of 493 questionnaires were finally collected, with an effective recovery rate of 98% (Figure 1). For latent profile analysis, simulation studies suggest that sample sizes of 300–500 are generally sufficient for stable class enumeration and parameter estimation, provided that each latent class contains at least 50–100 participants (15, 16). The final analytical sample of 493 haemodialysis nurses, with 217 and 276 participants in the two identified profiles, comfortably satisfied both logistic regression and LPA sample-size requirements. This study has been reviewed by the Ethics Committee of West China Hospital of Sichuan University with ethical approval number 2025(1259).

### Study instruments

#### Nurses’ general information questionnaire

The Nurses’ general information questionnaire was compiled by the research team, including age, gender, education, professional title, position, working years, marital status, etc.

#### Artificial Intelligence Literacy Scale (AILS)

This scale was developed by Wang et al. (17). The scale consisted of 4 dimensions and 12 items. The 4 dimensions are Awareness (Item 1, 2 and 3), Use (Item 4, 5 and 6), Evaluation (Item 7, 8 and 9), and Ethics (Item 10, 11 and 12). The Likert T7 scale was used. The options were “strongly agree, agree, somewhat agree, uncertain, somewhat disagree, disagree and strongly disagree,” corresponding to 7 to 1 points respectively, and there were three reverse items (Item 2, 5, and 11). The lowest score was 12 and the highest score was 84. Higher scores represent higher levels of Artificial Intelligence literacy. The Cronbach’s α of the scale was 0.83, respectively, which calculated in this study was 0.835.

#### The Nurse Innovative Behavior Scale (NIBS)

The scale was compiled by Bao et al. (18) based on the characteristics of Chinese nurses. The scale included three dimensions and 10 items: “Generating ideas,” “Obtaining support” and “Realizing ideas.” Likert five scoring method was used, with the 1–5 scores indicating “never,” “less,” “sometimes,” “often” and “frequently” respectively. The total score ranges from 10 to 50 points, with higher scores indicating higher innovative behavior. Its psychometric properties have been detailed in subsequent English-language publications (19, 20). The original scale demonstrated a Cronbach’s α of 0.879 and a content validity index of 0.910. In the present study, the Cronbach’s α for the total scale was 0.941, indicating excellent internal consistency. The higher alpha observed may reflect the relative homogeneity of the hemodialysis nurse sample and the strong inter-item correlations within this specific clinical context. The total score ranges from 10 to 50. For ease of interpretation and comparison with previous studies, the overall level of innovative behavior can also be expressed as the per-item mean score (total score divided by 10), which falls on the original 1∼5 response scale.

#### Data collection methods

The questionnaire was distributed and collected through the Internet platform “Questionnaire Star.”^1^ The head nurses or nurse managers of each participating dialysis center assisted in identifying eligible nurses according to the inclusion criteria and distributed the anonymous questionnaire link through departmental work groups or by providing a QR code. The questionnaire’s preface informed participants in detail of the study’s purpose, significance, content, and instructions for completion, and emphasized the confidentiality of all data. Informed consent was obtained from each participant. To prevent duplicate responses, the platform was configured to allow only one submission per account. The final dataset was exported without any IP addresses, and the research team had no access to any identifying information. Questionnaires with excessive response consistency, completion time less than 2 min (indicating rushed, inattentive responding) or contradictory answers were considered invalid and excluded from analysis.

#### Data analysis

The data were analyzed using SPSS27.0 and Mplus8.3 software. Measurement data following normal distribution were expressed as (mean ± SD), and comparison between groups was analyzed by *t*-test or one-way analysis of variance. Non-normal measurement data were expressed as median (interquartile range), and Kruskal-Wallis H test was used for comparison between groups. By examining the histograms, Q-Q plots, skewness and kurtosis values, it was determined that the total scores of NIBS and AILS were approximately normally distributed. Therefore, Pearson correlation coefficients were calculated to examine the associations between innovative behavior and AI literacy dimensions. As a robustness check, Spearman’s rank correlations were also computed and yielded substantively identical results. Prior to computing scale scores, the three reverse-worded items in the AILS were recoded so that higher scores consistently reflected higher AI literacy. All analyses used the recoded values. Taking the 10 items of the Nurses’ Innovative Behavior Scale as explicit variables, profiles from 1 to 4 were sequentially fitted from the 1-class benchmark model. Models with more than 3 profiles were initially attempted but failed to converge or resulted in a profile containing less than 5% of the total sample (indicating an unrepresentative and unstable class), and thus were not retained for further comparison. Consequently, the final analysis compared models with 1, 2, and 3 profiles. The evaluation indicators mainly included: Akaike Information Criteria (AIC), Bayesian Information Criteria (Bayesian Information Criteria, BIC) and adjusted Bayesian Information Criteria (aBIC). Smaller AIC, BIC and aBIC indicated better model fitting. Information X entropy (Entroy) was used to evaluate the classification accuracy of the model. The closer to 1, the higher the accuracy of the model. The likelihood ratio test (Lo-mendell-rubin, LMR) and Bootstrap likelihood ration test (BLRT) were used to compare the fitting effect of two adjacent models. *P* < 0.05 indicated that the model fitted well.

## Results

### Participant characteristics

A total of 503 questionnaires were distributed in this study, and 10 questionnaires that did not meet the requirements were excluded. 10 questionnaires were excluded for the following reasons: contradictory responses (*n* = 2, where age and years of work experience contradicted each other), or completion time < 2 min (*n* = 8). Finally, a total of 493 questionnaires were collected, with a recovery rate of 98%. The average age of hemodialysis specialist nurses participating in the survey was (34.62 ± 6.30) years old, and the proportion of females was 91.1%. The duration of nursing work was 12 (8.16) years, and the duration of hemodialysis nursing work was 7 (4.11) years. 87.6% of the nurses had bachelor’s degree or above.

### Latent profile analysis of nurse innovative behavior

**FIGURE 2:**
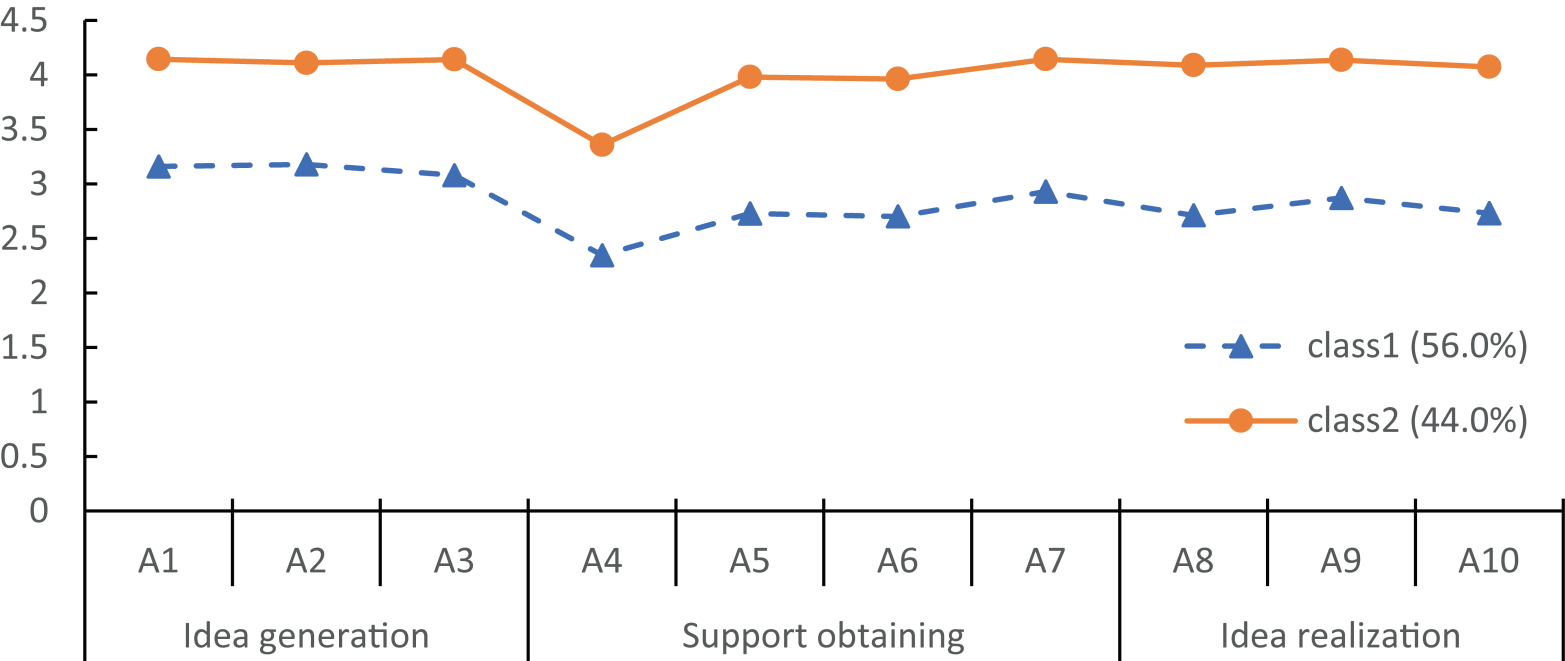
Two latent profiles of innovative behavior among hemodialysis nurses.

The overall per-item mean score of the NIBS was 3.35 ± 0.33 (equivalent to a total score of 33.5 ± 3.3), indicating a moderate level of innovative behavior. The latent profile analysis of hemodialysis nurses’ innovative behavior was performed using the 10 items of nurses’ innovative behavior scale as the explicit indicators, and the latent profile models from class 1 to class 3 were gradually fitted from the benchmark model of class 1, and the results are shown in Table 1. The AIC, BIC and aBIC of class 2 decreased most significantly, and the entropy value was 0.940, which was close to 1, and the *P*-values of LMR and BLRT were both < 0.01. Although the entropy value, LMR and BLRT of class 3 also met the requirements, the second class in class 3 accounted for only 8.5%, which was not conducive to later statistical analysis. Therefore, class 2 was identified as the best model in this study. According to the results of potential profile analysis, the line chart of the two profiles of innovative behavior of hemodialysis nurses on the 10 items showed that the proportion of nurses in Class 1 was 56.0% (276 cases), and the score of innovative behavior was (28.40 ± 4.55), which was relatively low. Class 2 accounted for 44.0% (217 cases), and the score of innovative behavior was (40.11 ± 4.36), which was higher. As shown in Figure 2, among all 10 items of NIBS, the score of class 2 was significantly higher than that of class 1 (28.40 ± 4.55 vs. 40.11 ± 4.36, *t* = −19.837, *P* < 0.001), which confirmed that class 2 demonstrated a significantly higher level of innovative behavior. Therefore, class 1 was labeled as “low innovative behavior-conservative” to reflect its lower score and tendency to maintain existing practices, while category 2 was labeled as “high innovative behavior-transcendence” according to Wang et al. (21), who found that nurses’ innovative behavior was positively correlated with self-transcendence. This naming highlights the proactive spirit of high innovators in actively seeking to transcend current norms., who found that innovative behavior among nurses is positively associated with self-transcendence. This naming highlights the aspirational nature of high innovators who actively seek to go beyond current routines.

**TABLE 1:** The latent profile fitting index of innovative behavior of hemodialysis nurses (*n* = 493).

Model	AIC	BIC	aBIC	LMR(P)	BLRT(P)	Entropy	Group size
Class 1	13024.299	13108.309	13044.829	–	–	–	1
**Class 2**	**10902.747**	**11032.963**	**10934.569**	**<0.001**	**<0.001**	**0.940**	**0.55984/0.44016**
Class 3	10087.594	10264.015	10130.707	<0.001	<0.001	0.962	0.51724/0.08519/0.39757

AIC, Akaike Information Criteria; BIC, Bayesian Information Criteria; aBIC, adjusted BIC; LMR, Lo-Mendell-Rubin test; BLRT, Bootstrap Likelihood Ratio Test. For the selected 2-class model, the average latent class probabilities for most likely class membership were 0.987 for Class 1 (“low innovative behavior–conservative”) and 0.977 for Class 2 (“high innovative behavior–transcendence”), indicating high classification accuracy. The 3-class model, the average latent class probabilities for most likely class membership were 0.991, 0.973, and 0.977. Bold font indicates the selected potential category that has been determined.

### Comparison of latent profiles of innovative behavior among hemodialysis nurses with different demographic

The results of univariate analysis showed that there were differences in gender, Number of years in the haemodialysis profession, Educational levels, Position of work, etc. between the two groups (*P* < 0.05). Details are provided in Table 2.

**TABLE 2:** Comparison of the potential characteristics of innovative behavior of hemodialysis nurses with different demographic characteristics.

Item	Total(n = 493)	Class 1(n = 276)	Class 2(n = 217)	*t*/*F*	*P*
Gender					
Male	44(8.9%)	18(6.5%)	26(12.0%)	4.455	**0.035**
Female	449(91.1%)	258(93.5%)	191(88.0%)		
**Age**	34.62 ± 6.30	34.42 ± 6.351	34.89 ± 6.234	0.827	0.409
**Number of years in the nursing profession**	12(8.16)	12(9.16)	12(8.17)	-0.725	0.469
**Number of years in the haemodialysis nursing profession**	7(4.11)	7(4.10)	8(4.12)	-2.015	**0.044**
Marital status					
Married	398(80.7%)	217(78.6%)	181(83.4%)	1.789	0.181
Others (unmarried, divorced, and widowed)	95(19.3%)	59(21.4%)	36(16.6%)		
Educational levels					
Associate degree	61(12.4%)	45(16.3%)	16(7.4%)	8.937	**0.003**
Bachelor’s degree and above	432(87.6%)	231(83.7%)	201(92.6%)		
Whether to obtain a specialist nurse certificate					
Yes	339(68.8%)	182(65.9%)	154(72.4%)	2.323	0.128
No	154(31.2%)	94(34.1%)	60(27.6%)		
Professional ranks and titles					
Registered nurse (RN) (China RN license)	24(4.9%)	18(6.5%)	6(2.8%)	4.962	0.175
Senior nurse	182(36.9%)	106(38.4%)	76(35.0%)		
Nurse in charge	253(51.3%)	134(48.6%)	119(54.8%)		
Associate chief nurse or above	34(6.9%)	18(6.5%)	16(7.4%)		
Position of work					
Head nurse	54(11%)	23(8.3%)	31(14.3%)	10.968	**0.004**
Team leader nurse	101(20.5%)	47(17.0%)	54(24.9%)		
Primary nurse	338(68.6%)	206(74.6%)	132(60.8%)		
Hospital level					
Tertiary hospital	364(73.8%)	196(71.0%)	168(77.4%)	2.738	0.254
Secondary hospital	74(15%)	47(17.0%)	27(12.4%)		
Private hospitals	55(11.2%)	33(12.0%)	22(11.2%)		
Whether to rotate night shift?					
Yes	330(66.9%)	193(69.9%)	137(63.1%)	2.534	0.111
No	163(33.1%)	83(30.1%)	80(36.9%)		
Do you think artificial intelligence will reduce the workload of nurses?					
Yes	379(76.9%)	200(72.5%)	179(82.5%)	**9.051**	**0.011**
No	37(7.5%)	21(7.6%)	16(7.4%)		
Be in doubt	77(15.6%)	55(19.9%)	22(10.1%)		
Will the nursing profession be hampered by greater use of AI technologies in the future?					
Yes	97(19.7%)	61(22.1%)	36(16.6%)	**8.449**	**0.015**
No	259(52.5%)	129(46.7%)	130(59.9%)		
Be in doubt	137(27.8%)	86(31.2%)	51(23.5%)		
Might the use of AI techniques in nursing practice lead to ethical concerns?					
Yes	122(24.7%)	72(26.1%)	50(23.0%)	**6.973**	**0.031**
No	177(34.7%)	82(29.7%)	89(41.0%)		
Be in doubt	200(40.6%)	122(44.2%)	78(359%)		
Do you think artificial intelligence technology should be included in the core curriculum of nursing education?					
Yes	312(63.3%)	155	157	**14.389**	**0.001**
No	47(9.5%)	29	18		
Be in doubt	134(27.2%)	92	42		

Bold font indicates that the *P* < 0.05.

### Correlation between innovative behavior and artificial intelligence literacy of hemodialysis nurses

As shown in Table 3, nurses in the “high innovative behavior–transcendence” profile (Class 2) scored significantly higher than those in the “low innovative behavior–conservative” profile (Class 1) on all AI literacy dimensions and on the total scale. Specifically, Class 2 exhibited higher awareness (16.42 ± 3.72 vs. 14.59 ± 2.59, *t* = −6.192, *P* < 0.001), usage (16.56 ± 3.18 vs. 14.54 ± 2.62, *t* = −7.586, *P* < 0.001), evaluation (16.61 ± 4.09 vs. 14.71 ± 2.89, *t* = −5.804, *P* < 0.001), and ethics scores (16.43 ± 3.28 vs. 14.86 ± 2.69, *t* = −5.706, *P* < 0.001). The total AI literacy score was also significantly greater in Class 2 (66.03 ± 11.89) than in Class 1 (58.70 ± 8.30; *t* = 8.055, *P* < 0.001). There was a significant weak positive correlation between the innovative behavior scale of hemodialysis nurses and the dimensions and total score of the Artificial Intelligence Literacy Scale (*P* < 0.001), as shown in Table 4.

**TABLE 3:** Comparison of artificial intelligence literacy scores of two potential categories of innovative behavior of nurses.

Variables	Class 1 (mean ± SD)	Class 2 (mean ± SD)	*t*	*P*
Awareness	14.59 ± 2.59	16.42 ± 3.72	−6.192	**0.000**
Use	14.54 ± 2.62	16.56 ± 3.18	−7.586	**0.000**
Evaluation	14.71 ± 2.89	16.61 ± 4.09	−5.804	**0.000**
Ethics	14.86 ± 2.69	16.43 ± 3.28	−5.706	**0.000**
Total score	58.696 ± 8.30	66.032 ± 11.89	8.055	**0.000**

Bold font indicates that the *P* < 0.05.

**TABLE 4:** The correlation between nurses’ innovative behavior and artificial intelligence literacy (*n* = 493).

Variables	Dimensions	1	2	3	4	5	6	7	8	9
AILS	1. Awareness	1	1	1	1	1	1	1	1	1
	2. Use	0.732**								
	3. Evaluation	0.526**	0.592**							
	4. Ethics	0.538**	0.577**	0.443**						
	5. Total AILS	0.844**	0.870**	0.793**	0.752**					
NIBS	6. Idea generation	0.345**	0.408**	0.224**	0.338**	0.389**				
	7. Support obtaining	0.212**	0.248**	0.255**	0.159**	0.262**	0.594**			
	8. Idea realization	0.297**	0.337**	0.283**	0.245**	0.344**	0.661**	0.778**		
	9. Total NIBS	0.325**	0.370**	0.294**	0.260**	0.373**	0.816**	0.904**	0.906**	

AIAS, Artificial Intelligence Anxiety Scale; NIBS, Nurse Innovative Behavior Scale.

*p < 0 .05.

**p <0 .01.

### Multivariate analysis of the potential profile of innovative behavior of hemodialysis nurses

The potential categories of innovative behavior among hemodialysis nurses were used as the dependent variable. The statistically significant variables in the univariate analysis were used as independent variables, and the first item of the categorical variable was used as a reference for binary Logistic regression analysis. As shown in Table 5, artificial intelligence literacy score was a significant independent predictor (OR = 1.075, 95% CI: 1.053∼1.096, *p* < 0.001). Nurses with a bachelor’s degree or above were less likely to belong to the low innovative behavior profile compared to those with an associate degree (OR = 0.500, 95% CI: 0.264∼0.949, *p* = 0.034). Team leader nurses had significantly higher odds of being in the high innovative behavior profile compared to head nurses (OR = 2.074, 95% CI: 1.115∼3.859, *p* = 0.021). The model demonstrated acceptable fit (Hosmer–Lemeshow test, *P* = 0.077), and multicollinearity was negligible (all VIF < 1.02).

**TABLE 5:** Multivariate analysis of the potential profile of innovative behavior of hemodialysis nurses.

Variables	Model 1		Model 2	
	OR(95%CI)	*P*	OR(95%CI)	*P*
AILS	1.076(1.055∼1.098)	0.000	1.075(1.053∼1.096)	0.000
Educational levels				
Associate degree			Reference	0.034
Bachelor’s degree and above			0.500(0.264∼0.949)	
Position of work				
Head nurse			Reference	0.021
Team leader nurse			2.074(1.115∼3.859)	
Primary nurse			1.600(0.991∼2.582)	0.054

OR, odds ratio; CI, confidence interval. The dependent variable was latent profile membership (1 = low innovative behavior–conservative (reference category) and 2 = high innovative behavior–transcendence). Model 1: unadjusted. Model 2: adjusted for educational level and position of work. Hosmer–Lemeshow test: χ^2^ = 14.202, df = 8, *P* = 0.077. All independent variables showed VIF values ranging from 1.002 to 1.018 and tolerance values from 0.983 to 0.998, confirming the absence of multicollinearity.

## Discussion

In the present study, the overall per-item mean NIBS score of haemodialysis nurses was 3.35 ± 0.33 (on the 1–5 scale), which is comparable to the per-item means reported in previous studies using the same instrument: 3.08 ± 0.67 by Zhou et al., suggesting that the innovative behavior of hemodialysis nurses in China is still at a medium level at this stage (11). However, compared with the results of Fu et al. (19) (2.81 ± 0.66), the average score of innovative behavior of nurses in this study was higher. This may be related to the 42.34% of secondary hospitals in the survey by Fu et al. However, in our survey, the proportion of tertiary hospitals is about 73.8%. In addition, the survey population was nurses engaged in hemodialysis, and Fu et al. surveyed nurses in traditional Chinese medicine hospitals. The lack of objective evaluation criteria for traditional Chinese medicine nursing skills may affect the ability of traditional Chinese medicine nursing business research and the implementation of innovative ideas. Nurses engaged in hemodialysis not only need to provide specialized care during the treatment of dialysis patients, but also need to master the operation methods and alarm handling methods of various types of hemodialysis machines (22). Under such a high-intensity and high-demand working environment, it is necessary for nurses to find more learning ways and innovative thinking so as to stimulate higher innovative behavior and ability of hemodialysis nurses.

This study identified two potential characteristics of the innovative behavior of hemodialysis nurses. They are “low innovation behavior-conservative” and “high innovation behavior-transcendence.” The two latent categories accounted for 56 and 44%, respectively. There were statistical differences in education level and job position between the two categories, and hemodialysis found a significant positive correlation between innovative behavior of nurses and artificial intelligence literacy. That is, the Artificial Intelligence literacy level of “high innovative behavior-transcendence” was significantly higher than that of “low innovative behavior-conservative.” Although there is no potential profile analysis results of innovative behaviors of hemodialysis nurses, Li et al. (20) and Jiwen et al. (23) investigated the innovative behaviors of clinical nurses and identified three potential characteristics. The identification of only two latent profiles in the present sample, in contrast to the three profiles reported in general clinical nurses, deserves particular attention. One possible explanation lies in the professional homogeneity of haemodialysis nursing. Haemodialysis nurses work in a highly specialized environment with standardized protocols for machine operation, complication management, and patient education (22), which may naturally restrict the range of innovative expression to a dichotomy between “maintaining the status quo” and “actively transcending routines.” Another contributing factor may be sample composition. Our sample was predominantly from tertiary hospitals (73.8%), where innovation culture is more robust, potentially compressing a middle or extremely low subgroup. In contrast, Fu et al. included 42.3% secondary hospitals, which may have allowed a distinct “low” group to emerge. Thus, the two-class solution likely reflects the unique attributes of haemodialysis nurses and their work contexts, rather than a statistical artifact. Future multi-center studies with wider hospital-level representation are warranted to confirm whether three profiles re-emerge in more heterogeneous dialysis settings.

Position of work was also mentioned in their results as an important factor affecting innovative behavior, similar to the results of the present study. That is, nurses in the “high innovative behavior-transcendence” group had higher positions, mainly head nurses and team leader nurse. Such nurses may have high demands on themselves and are willing to develop upward and surpass themselves. This suggests that nurses in high positions are more likely to make effective use of resources and understand new technologies and methods in the nursing field in real time so as to improve their innovation ability. Therefore, nursing managers can consider increasing the promotion opportunities and positions of nurses to improve the innovative behavior of hemodialysis nurses. In this study, hemodialysis nurses with bachelor’s degree or above were more likely to have high innovative behaviors, which is similar to the results of previous studies (23, 24). The research results of Jiwen et al. suggested that highly educated nurses scored higher in the dimension of generating ideas (23). Emiralioğlu and Sönmez (24) suggested that as the level of education increases, the likelihood of nurses to exhibit innovative behaviors also increases. The process of education upgrading can obtain new knowledge and stimulate thinking. Therefore, nursing managers engaged in hemodialysis can stimulate the level of innovative behavior by encouraging nurses to improve their education.

This study shows that there is a correlation between the innovative behavior of hemodialysis nurses and the level of artificial intelligence literacy. The strength of the correlation between artificial intelligence literacy and innovative behavior (r = 0.373, P < 0.001) indicates a moderate positive correlation, suggesting that artificial intelligence literacy is a meaningful but not the sole contributing factor to innovative behavior. According to the social cognitive theory (25), individuals with higher artificial intelligence literacy may have stronger technological self-efficacy, enabling them to try new solutions and embrace change. This explanation aligns with the AILS (Artificial Intelligence Literacy Scale) dimension: “Awareness” and “Use” reflect active mastery experiences, while “Evaluation” and “Ethics” involve reflection and moral agency - both of which are crucial for transforming ideas into implementation. Moreover, the self-determination theory (26) posits that competence and autonomy are core psychological needs, and artificial intelligence literacy can fulfill these needs by enabling nurses to independently access evidence-based resources and creatively solve problems. This moderate correlation also indicates that artificial intelligence literacy alone is not sufficient to drive innovation; organizational culture, leadership support, and intrinsic motivation remain indispensable. Future research should test the mediating model and examine its interaction effects with environmental factors.

Hemodialysis nurses with high innovation behavior also have higher scores in all dimensions of artificial intelligence literacy. At present, there is no relevant research on the relationship between nurses’ innovative behavior and artificial intelligence literacy, but the existing research on nursing students shows that the stronger the artificial intelligence literacy and innovative behavior ability, the higher the self-efficacy(27). Artificial intelligence literacy is defined as the ability to be aware of and understand artificial intelligence technologies in practical applications, to be proficient in using artificial intelligence applications and products to accomplish tasks, to analyze, select, and critically evaluate the data and information provided by artificial intelligence, and to establish a certain sense of responsibility and respect for the rights and obligations of peers (17). Therefore, the four dimensions of the Artificial intelligence literacy scale are awareness, usage, evaluation and ethics. Nurses with high innovation behavior are more likely to have ideas, and they will obtain relevant support and take action while generating ideas. Artificial intelligence is a very suitable way to solve the above behaviors. Due to the development of The Times, the continuous changes of patients’ conditions and the iterative update of therapeutic instruments, hemodialysis nurses need to constantly use artificial intelligence to learn new technologies, solve problems and improve their self-ability. Therefore, there is a certain interaction between innovative behavior and artificial intelligence literacy, which may be the reason for the correlation between innovative behavior and artificial intelligence literacy of hemodialysis nurses. The higher the level of AI literacy, the more likely nurses were to exhibit high innovative behavior profiles.

## Limitations

Several limitations of this study should be considered when interpreting the findings. First, the cross-sectional design precludes causal inference; the observed association between AI literacy and innovative behavior cannot be interpreted as a cause-effect relationship, and longitudinal studies are needed to establish temporality. Second, convenience sampling was used, and although participants were recruited from multiple cities in Sichuan Province, China, the sample may not be fully representative of hemodialysis nurses in other regions or institution types. Furthermore, the research team is affiliated with a single institution (West China Hospital, Sichuan University), which, while the study received multi-center ethical approval from this institution, could introduce an institutional cultural lens in data interpretation. Third, all variables were measured via self-reported questionnaires, which are subject to social desirability and common method bias. Specifically, the Artificial Intelligence Literacy Scale (AILS) assesses perceived competence rather than objectively demonstrated AI skills; future research should incorporate performance-based measures of AI literacy to validate these findings. Fourth, the labeling of the latent profiles as “low innovative behavior–conservative” and “high innovative behavior–transcendence” was guided by a prior correlation study rather than qualitative validation within the current sample, and thus the labels should be viewed as interpretive. Fifth, although the sample size met the requirements for the planned analyses, the relatively small number of participants in certain subgroups (e.g., male nurses, associate degree holders) limited statistical power for subgroup comparisons. Finally, this study did not measure organizational-level factors such as innovation climate or leadership support, which may confound or moderate the AI literacy–innovation relationship. Future research should adopt longitudinal, multi-center, mixed-method designs to address these limitations.

## Conclusion

The innovative behavior of hemodialysis nurses is at a medium level, which can be divided into two potential categories: “low innovative behavior-conservative” and “high innovative behavior-transcendental.” Artificial intelligence literacy is a positive factor affecting its innovative behavior. At the same time, improving the education of hemodialysis nurses and increasing their opportunities for promotion are more likely to improve their innovative behavior.

## Data Availability

The original contributions presented in this study are included in this article/Supplementary material, further inquiries can be directed to the corresponding authors.

## Footnotes

1
https://www.wjx.cn/
